# Diagnostic delay in adult inflammatory bowel disease: A systematic review

**DOI:** 10.1007/s12664-022-01303-x

**Published:** 2023-01-30

**Authors:** Eleanor Cross, Benjamin Saunders, Adam D. Farmer, James A. Prior

**Affiliations:** 1 School of Medicine, Keele University, Keele, Staffordshire, ST5 5BG UK; 2 University of North Midlands (UHNM) NHS Trust, Stoke-On-Trent, UK; 3 Department of Gastroenterology, University Hospital of North Midlands (UHNM) NHS Trust, Stoke-On-Trent, UK; 4 Midlands Partnership NHS Foundation Trust, Trust Headquarters, St. George’s Hospital, Stafford, UK

**Keywords:** Crohn’s disease, Diagnostic delay, Inflammatory bowel disease, Ulcerative colitis

## Abstract

**Background:**

The extent of diagnostic delay in inflammatory bowel disease (IBD) is incompletely understood. We aimed to understand the extent of diagnostic delay of IBD in adults and identify associations between patient or healthcare characteristics and length of delay.

**Methods:**

Articles were sourced from EMBASE, Medline and CINAHL from inception to April 2021. Inclusion criteria were adult cohorts (18 ≥ years old) reporting median time periods between onset of symptoms for Crohn’s disease (CD), ulcerative colitis (UC) or IBD (i.e. CD and UC together) and a final diagnosis (diagnostic delay). Narrative synthesis was used to examine the extent of diagnostic delay and characteristics associated with delay. Sensitivity analysis was applied by the removal of outliers.

**Results:**

Thirty-one articles reporting median diagnostic delay for IBD, CD or UC were included. After sensitivity analysis, the majority of IBD studies (7 of 8) reported a median delay of between 2 and 5.3 months. From the studies examining median delay in UC, three-quarters (12 of 16) reported a delay between 2 and 6 months. In contrast, three-quarters of the CD studies (17 of 23) reported a delay of between 2 and 12 months. No characteristic had been examined enough to understand their role in diagnostic delay in these populations.

**Conclusions:**

This systematic review provides robust insight into the extent of diagnostic delay in IBD and suggests further intervention is needed to reduce delay in CD particularly. Furthermore, our findings provide a benchmark value range for diagnostic delay, which such future work can be measured against.

**Supplementary Information:**

The online version contains supplementary material available at 10.1007/s12664-022-01303-x.

Bullet points of the study highlights ***What is already known?***

- Diagnosis of inflammatory bowel disease (IBD)﻿ is frequently delayed.
- Reported extent of delay is varied, ranging from a few months to several years.
- It remains unclear whether certain patient groups are more vulnerable to delay.

***What is new in this study?***

- The provisions of tighter benchmarks of the extent of diagnostic delay in IBD.
- Examination of the role of specific characteristics (disease type, age of onset) on diagnostic delay of IBD.

***What are the future clinical and research implications of the study findings?***

- Impact of newer diagnostic tests like fecal calprotectin on the delay in IBD diagnosis.
- More research is required into the role of patient characteristics on the extent of diagnostic delay.

## Introduction

Inflammatory bowel disease (IBD) is a chronic inflammatory condition affecting the gastrointestinal tract, the most prevalent examples being Crohn’s disease (CD) and ulcerative colitis (UC) [1]. Approximately 6.8 million cases of IBD were reported globally in 2017, with nearly a quarter of these cases in North America, resulting in a significant personal and societal burden [2].

The clinical presentation of IBD is variable, but can include change in bowel habit, rectal bleeding, fatigue and weight loss [3]. In addition, approximately 25% to 40% of individuals with IBD display extraintestinal manifestations, which include arthritis, axial spondyloarthritis, uveitis, erythema nodosum and primary sclerosing cholangitis [4]. The range of such presenting symptoms can make it challenging for clinicians to promptly identify patients with IBD, and symptoms can be attributed to other conditions such as irritable bowel syndrome, all of which can lead to diagnostic delay [5]. Such delays may be further contributed to by factors such as patient demographics, geographical location and the presence of extraintestinal manifestations [6–9]. For example, in affected countries, symptoms of IBD may be mistaken for abdominal tuberculosis [10]. In cases of diagnostic delay, adverse impacts on clinical outcomes can occur, including an increased need for subsequent surgical intervention and poor response to medical therapy [11, 12].

Recent reports suggest the average diagnostic delay of IBD can range anywhere from 2 months to 8 years [5, 13] and that delays in the diagnosis of CD are longer than for UC [6, 12, 14]. This wide variation reported in the differences in the delay experienced by patients, means the true extent of the problem of delay remains unclear. The aim of this systematic review was to provide a clearer benchmark range for the extent of diagnostic delay, as well as providing information on any characteristics that may be associated with delay.

## Methods

### Data sources and searches

We conducted a systematic review by searching the medical literature databases of Medline, EMBASE and CINAHL from their inception to April 2021, using a combination of free-text and medical subject headings (MeSH) terms, or equivalents from each database (Supplementary Table 1). Search terms were devised for IBD and diagnostic delay using existing systematic reviews that explored other aspects of IBD, and reviews investigating diagnostic delay in other long-term conditions [15–17]. Preferred Reporting Items for Systematic Reviews and Meta-Analyses (PRISMA) guidelines were followed during the completion of this systematic review and the protocol was logged with International Prospective Register of Systematic Reviews (PROSPERO) (registration number: CRD42018108886).

### Study selection

The inclusion criteria were developed using the Population, Intervention, Comparison, Outcomes and Study (PICOS) framework [18]. The included populations were adults aged 18 years or older with a confirmed diagnosis of IBD, CD or UC. Studies with adult populations which included a proportion of participants under 18 years old were retained if the mean age implied the population was largely formed from participants above 18 years old. Studies also had to include the primary outcome of interest, a reported average time period of diagnostic delay for IBD, CD or UC from symptom onset to final diagnosis.

Studies were excluded if they examined delay, but did not report data related to the extent of, or a characteristic related to, the time period of diagnostic delay (i.e. defined as wrong outcomes) or their design included case studies or case series with less than ten participants, literature reviews, systematic reviews or conference abstracts. There were no restrictions on time of publication, as databases were searched from inception. Articles in languages other than English were included, individuals who spoke the language were then requested to make initial assessments and then, if necessary, quality appraise and extract pertinent data, with Google Translate also being used if necessary.

Once the database searches had been undertaken using the reported criteria, the reference manager software Mendeley was used to remove duplicates (Version 1.16.1, Mendeley Ltd., London, UK). The authors E. C. and J. A. P. then completed an independent title and abstract review of 50% of the initially identified articles each. Articles that progressed to abstract review underwent a second review, where E. C. reviewed the abstracts initially reviewed by J. A. P. and vice versa. These included abstracts then underwent a full-text review, with E. C. independently reviewing all full texts and the remaining three authors (J. A. P., B. S., A. D. F.) reviewing a proportion of full texts each. Throughout the review process, disagreements between reviewers were resolved through discussion and by consulting an arbitrating author (BS).

### Data extraction and analysis

Data extraction of included articles was completed by two reviewers (E. C., J. A. P.). The primary outcome of interest extracted was the reported average time period of diagnostic delay of IBD (for articles that did not distinguish between CD and UC), CD or UC, from symptom onset to diagnosis. Average data regarding diagnostic delay could be reported as mean or median values, with both being recorded along with their accompanying estimate of accuracy (standard deviation or interquartile range respectively). However, due to the typical non-normality of mean diagnostic delay data, only median values were used in this analysis [19]. This allowed for data which was more representative of the average delay actually experienced by patient samples, though removed the possibility to pool data from all included articles using meta-analysis techniques. The unit of time that articles used to report diagnostic delay varied from days to years; therefore, delay data were converted into months to allow comparability of the data.

Additional data extracted included lead author, year of publication, time period of participant recruitment, gender, country and mean sample age. Finally, information (where reported) on any specific characteristics (e.g. demographics, symptoms) and their association with reported diagnostic delay were identified in the articles and extracted. Regarding this characteristic data, no restriction was placed on the measure of central tendency here, but rather the focus was on whether differences in delay were experienced across comparator samples (i.e. extent of diagnostic delay in males vs. females). The data from the included articles were examined using narrative synthesis. In the instance of outlier data, sensitivity analysis was performed, resulting in the exclusion of related studies.

### Quality appraisal

A modified Newcastle–Ottawa Score (NOS) was used for quality appraising the included articles, carried out by E. C. and J. A. P. An adapted version of the NOS for cohort studies and for cross-sectional studies were used [20]. The ‘Selection of participants’ and ‘Measurement of outcome’ were the two criteria of the NOS used to assess quality of articles. The representativeness of studies was assessed based on the geographical spread of recruitment, as well as variation in population characteristics.

## Results

### Search results

**Fig. 1:**
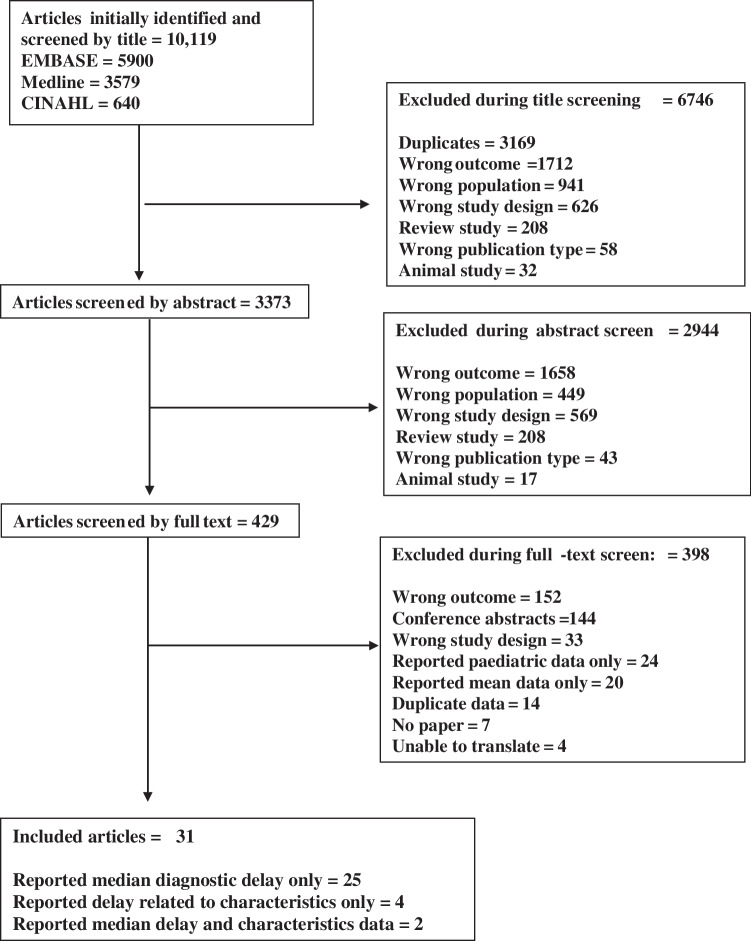
Preferred Reporting Items for Systematic Reviews and Meta-Analyses (PRISMA) flowchart of the screening process used to identify the included articles

A total of 10,119 unique articles were identified from the three selected databases and underwent title review. Following the exclusion of 6746 articles based upon title, 3373 articles underwent abstract review. From these, 429 articles were reviewed in full, leaving a final 31 articles that reported median data to be included for narrative synthesis (see PRISMA flowchart, below [Fig. 1]).

### Study characteristics

Of the 31 articles included in the review, 23 were cohort studies, of which 12 were retrospective [6, 12–14, 21–28], eight were prospective cohorts [9, 29–35] and three combined both methods [36–38]. Eight articles used a cross-sectional study design [5, 7, 8, 11, 39–42]. Nineteen studies were conducted in Europe, seven in Asia, four in North America and one in South America. Twenty articles were based in primary or secondary care, four in tertiary care and five from population registries. Two articles did not report the source from which the sample was drawn.

The majority of articles typically documented the criteria used to diagnose IBD in participants, as a combination of clinical features, radiologic and histologic findings. Formal diagnostic criteria used by articles included Lennard–Jones, European Crohn’s and Colitis Organization (ECCO) and Copenhagen diagnostic criteria. Nine articles did not outline the use of any diagnostic criteria. The characteristics of each study included in the review have been summarized in Table 1.

**Table 1:** The characteristics of the studies included in the systematic review (*n* = 31)

Author, year	Country	Study design	Recruitment period	Healthcare setting	Condition	Diagnostic criteria for IBD
Kyle, 1971 [39]	Scotland	Cross-sectional	1955–1969	Not reported	CD	History and examination findings, radiology/endoscopy
Lind et al., 1985 [29]	Norway	Prospective cohort	1975–1979	Secondary	CD	Standardised investigational program (colonoscopy/gastroscopy. biopsy, barium enema)
Foxworthy and Wilson, 1985 [30]*	USA	Prospective cohort	1975–1983	Secondary	CD	Clinical presentation/course, radiologic/histologic/laparotomy appearance
Harper et al., 1986 [8]*	USA	Cross-sectional	1983–1984	Secondary	CD	Typical symptoms/findings, radiologic/endoscopic/operative features
Langholz et al., 1991 [31]	Denmark	Prospective cohort	1962–1987	Secondary	UC	Copenhagen criteria
Munkholm et al., 1992 [32]	Denmark	Prospective cohort	1962–1987	Secondary	CD	Copenhagen criteria
Timmer et al., 1999 [33]	Germany	Prospective cohort	1991–1995	Secondary	CD	Case definition criteria for definite or probable CD
Yang et al., 2000 [36]	South Korea	Retrospective and prospective cohort	1986–1977	Secondary	UC	Clinical, sigmoidoscopy, histological/cytological, radiologic/endoscopic features
Burgmann et al., 2006 [5]	Canada	Cross-sectional	2004–2005	Manitoba IBD registry	CD	ICD codes
					UC	ICD codes
Albert et al., 2008 [40]	Germany	Cross-sectional	2005–2007	Patient organisation	CD	Not reported
Romberg-Camps et al., 2009 [34]	Netherlands	Prospective cohort	1991–2002	Population registry	CD	Lennard–Jones criteria
					UC	Continuous mucosal inflammation without granuloma, affecting rectum + /some/all of the colon in continuity with rectum
Guariso et sl., 2010 [37]	Italy	Retrospective and prospective cohort	1994–2008	Tertiary	IBD	Clinical, radiological, endoscopic + histologic findings
					CD	Clinical, radiological, endoscopic + histologic findings
					UC	Clinical, radiological, endoscopic + histologic findings
Vavricka et al., 2012 [6]	Switzerland	Retrospective cohort	2006–2009	Population registry	CD	Not reported
					UC	Not reported
Burisch et al., 2014 [9]*	Europe	Prospective cohort	Jan 2010–Dec 2010	Secondary	CD	Copenhagen diagnostic criteria
					UC	Copenhagen diagnostic criteria
Pellino et al,, 2015 [11]	Italy	Cross-sectional	2000–2009	Secondary	CD	Accepted ECCO criteria
Li et al., 2015 [24]	China	Retrospective cohort	2010–2014	Secondary	CD	Colonoscopy, enteroscopy, capsule endoscopy, histopathology, haematology
Maconi et al., 2015 [22]	Italy	Retrospective cohort	2012–2013	Primary and secondary	CD	Not reported
Basaranoglu et al., 2015 [13]	Turkey	Retrospective cohort	1995–2007	Tertiary	CD	Clinical, radioscopic, endoscopic, histologic findings
					UC	Not reported
Lin et al., 2016 [25]*	Taiwan	Retrospective cohort	1991–2014	Not reported	UC	Not reported
Lee et al., 2017 [12]	South Korea	Retrospective cohort	2000–2015	Secondary	CD	Not reported
						Not reported
Cantoro et al., 2017 [21]	Italy	Retrospective cohort	1955–2014	Secondary	IBD	Not reported
Nguyen et al., 2017 [14]	USA	Retrospective cohort	2008–2015	Tertiary	CD	Not reported
					UC	Not reported
Nahon et al., 2018 [38]	France	Retrospective and prospective cohort	2002–2015	Secondary	CD	Colonoscopy or radiologic examination which strongly suggested IBD
					UC	
Irving et al., 2018 [41]	Finland, Italy, France, Canada, Germany, UK, Spain and Sweden	Cross-sectional	2013–2014	General population	CD	Not reported
					UC	Not reported
Szanto et al., 2018 [23]	Hungary	Retrospective cohort	2007–2015	Tertiary	CD	Lennard–Jones and accepted ECCO criteria
					UC	Lennard–Jones and accepted ECCO criteria
Banerjee et al., 2018 [26]	India	Retrospective cohort	Not reported	Secondary	CD	Not reported (paediatric CD = Porto criteria)
Kang et al., 2019 [27]	South Korea	Retrospective cohort	2006–2016	Secondary	UC	Clinical, radiological, endoscopic and pathologic findings
Novacek et al., 2019 [7]	Austria	Cross-sectional	2014–2015	Secondary	CD	ECCO criteria
					UC	ECCO criteria
Walker et al., 2020 [28]	UK	Retrospective cohort		Primary and secondary	CD	ECCO-ESGAR criteria
					UC	ECCO-ESGAR criteria
Gomes et al., 2021 [42]	Brazil	Cross-sectional	Not reported	Secondary	CD	Clinical, laboratory, endoscopic and histologic findings
					UC	Clinical, laboratory, endoscopic and histologic findings
Chaparro et al., 2021 [35]	Spain	Prospective cohort	2017	Secondary	CD	ECCO criteria
					UC	ECCO criteria

*IBD* inflammatory bowel disease, *CD* Crohn’s disease, *UC* ulcerative colitis, *ICD* International Classification of Diseases, *ECCO* European Crohn’s and Colitis Organisation. *Studies providing diagnostic delay data for characteristic comparisons only

### Quality appraisal

Of the 23 cohort studies, eleven articles were deemed to be ‘truly representative’, 11 ‘somewhat representative’ and one sampled from a ‘selected group of users’. For the eight cross-sectional studies, four articles were ‘truly representative’, three ‘somewhat representative’, one sampled from a ‘selected group of users’ and one ‘did not report the sampling strategy’. Ascertainment of exposure (i.e. diagnosis of IBD) was determined by assessing ‘secure records’ in all cohort studies. For the eight cross-sectional studies, three studies used a ‘validated measurement tool’ to ascertain exposure, in three studies a ‘tool was available and described’ and two studies provided ‘no description’ of how participants with IBD were identified. Assessment of outcome, which in this systematic review was diagnostic delay, was done by ‘record linkage’ in 17 cohort studies and the remaining six by ‘self-report’. Two cross-sectional studies used ‘record linkage’, four used ‘self-reporting’ and two studies provided ‘no description’ for the assessment of outcome (Supplementary Table 2).

### Extent of diagnostic delay

There were 11,597 participants providing median data related to IBD, with 4269 participants originating from studies that did not distinguish between CD or UC. A total of 13,998 participants provided data related to CD and 12,895 provided data relating to UC. Median values of diagnostic delay of IBD were provided by nine articles and ranged from 2 to 96 months. Median delay of CD was provided by 24 articles, ranging from 2 to 84 months and 17 articles reported median delays of 2 to 114 months from initial symptoms to final diagnosis of UC (Table 2).

**Table 2:** Median diagnostic delay (months) for inflammatory bowel disease, Crohn’s disease and ulcerative colitis ordered from shortest to longest delay

Lead author	Year	Country	Sample size ( *n*)	% male	Mean age (SD)/[range]	Diagnostic delay	IQR	Range
Inflammatory bowel disease (IBD)								
Basaranoglu et al. [13]	2015	Turkey	282 CD-98 UC-184	64.2	40.1 (14.7)	2	0*–*18	
Cantoro et al. [21]	2017	Italy	3392 CD-1537 UC-1855	53.1	Not reported	3	(0*–*13)	
Romberg-Camps et al. [34]	2009	Netherlands	1187 CD-476 UC-630 IC-81	48.9	CD-34 [5–79] UC-42 [8–84] IC-42 [13–77]	3		0*–*480
Guariso et al. [37]	2010	Italy	179 CD-65 UC-99 IBD-U-15	-	29.1 [18–40]	3		0*–*135
Chaparro et al. [35]	2021	Spain	3611	52.8	42^†^ (30–55)	3		1*–*9
Szanto et al. [23]	2018	Hungary	911 CD-428 UC-483	46.3	Not reported	3.6	(0*–*9.6)	
Novacek et al. [7]	2019	Austria	1265 CD-830 UC-435	49.4	40 (31–52)* [18–87]	5.3	(2.3*–*16.4)	
Gomes et al. [42]	2021	Brazil	658	44.6	44.2	13		5*–*38
Burgmann et al. [5]	*2006*	*Canada*	*112* *CD-65* *UC-42* *Proctitis-3* *IC-2*	*42.9*	*38 (12.9) [16*–*77]*	*96*		
Crohn’s disease (CD)								
Basaranoglu et al., [13]	2015	Turkey	98	52.0	37.8 (13.5) [17*–*79]	2	-	[0–18]
Guariso et al., [37]	2010	Italy	65	-	-	4	-	0*–*88
Timmer et al., [33]	1999	Germany	132	25	31 (23*–*47)*	5	-	
Romberg-Camps et al. [34]	2009	Netherlands	476	39.3	34 [5*–*79]	5	-	[0*–*360]
Chaparro et al., [35]	2021	Spain	1647	50.2	41^†^ (28*–*54)	5	-	1*–*15
Nahon et al., [38]	2018	France	638			5	-	-
Kyle [39]	1971	Scotland	175	38.3	Not reported	6	-	-
Lee et al. [7]	2017	South Korea	165	76.4	28.2 (13.8)	6.2	(1.5*–*21.5)	-
Novacek et al., [7]	2019	Austria	830	48.1	40 (31–52)*	6.4	(2.3*–*23.4)	-
Cantoro et al., [21]	2017	Italy	1537	53.1	Not Reported	7.1	-	-
Walker et al., [28]	2020	UK	94	-	-	7.6	3.1*–*16.0	0*–*112
Maconi et al., [22]	2015	Italy	83	41	35.2 (14.5)	8	(3*–*27)	[0*–*324]
Vavricka et al., [6]	2012	Switzerland	932	46.8	41 (15) [16*–*88]	9	(3*–*24)	-
Nguyen et al., [14]	2017	USA	110	41.0	35 (17)	9.5	(3.8*–*25.6)	-
Li et al., [24]	2015	China	343	70.0	29.5 (12.0)	10	(2*–*34)	-
Pellino et al., [11]	2015	Italy	361	Not reported	32.54 (14.47)	11	-	[1*–*163]
Irving et al., [41]	2018	Finland, Italy, France, Canada, Germany, UK, Spain and Sweden	4097	Not reported	Not reported	12	-	0*–*564
Albert et al., [40]	2008	Germany	112	34.8	28 (8.0) F 31 (11.5) M	13	-	[0*–*281]
Banerjee et al., [26]	2018	India	720	60.3	32 (18–50)*	18	(6*–*36)	-
Gomes et al., [42]	2021	Brazil	303	49.8	32	20	6.5*–*48	-
Lind et al., [29]	1985	Norway	214	56.1	24 [7*–*63] 22*	24		
Szanto et al., [23]	2018	Hungary	428	45.3	26.6 (11.3)	25.2	0*–*103.2	
Munkholm et al., [32]	1992	Denmark	373	42.1	32.5, 8*–*84	26.4		0*–*324
Burgmann et al., [5]	*2006*	*Canada*	*65*	*33.8*	*38 (12.9) [16–77]*	*84*		
Ulcerative colitis (UC)								
Guariso et al., [12]	2010	Italy	99	-	-	2	-	0*–*135
Basaranoglu et al., [13]	2015	Turkey	98	52.0	37.8 (13.5) [17*–*79]	2		0*–*15
Cantoro et al., [21]	2017	Italy	1855	53.1	Not reported	2		
Chaparro et al., [35]	2021	Spain	1964	50.1	46^†^ (34*–*57)	2		1*–*5
Kang et al., [27]	2019	South Korea	551	55.7	40.56 (16.11)	2.3		
Romberg-Camps et al., [34]	2009	Netherlands	476	39.3	34, 5*–*79	3		0*–*480
Nahon et al., [38]	2018	France	272			3		
Nguyen et al., [14]	2017	USA	110	41.0	38 (17)	3.1	1.1*–*9.6	
Walker et al., [28]	2020	UK	195	-	-	3.3	1.9*–*7.3	0*–*65
Novacek et al., [7]	2019	Austria	830	48.1	40 (31–52)*	3.4	1.4*–*10.3	
Vavricka et al., [6]	2012	Switzerland	932	46.8	41 (15) [16*–*88]	4	1*–*12	
Yang et al., [36]	2000	Korea	94	47.9	35* [8*–*68]	6		1*–*120
Gomes et al., [42]	2021	Brazil	355	38.3	35.3	11	4*–*29	
Irving et al., [41]	2018	Finland, Italy, France, Canada, Germany, UK, Spain and Sweden	3410	Not reported	Not reported	12		0*–*552
Langholz et al., [31]	1991	Denmark	1161	46.7	34 [2*–*88]	12		0*–*444
Szanto et al., [23]	2018	Hungary	428	45.3	26.6 (11.3) 30.9 (12.5)	55.2	0*–*123.6	
Burgmann et al., [5]	*2006*	*Canada*	*65*	*33.8*	*38 (12.9) [16–77]*	*114*		

*IC* indeterminate colitis, *IBD-U* unclassified inflammatory bowel disease, *SD* standard deviation; *IQR*, interquartile range. *Median age reported with interquartile range, Studies in italic indicates outliers. ^†^Median age provided

Through the sensitivity analysis, one article was excluded due to consistently being an outlier across the three condition groups. Burgmann et al. [5] reported median delays of approximately 60–75 months longer than the next closest study across the three analyses. After exclusion of this outlier, the median diagnostic delay of IBD across the remaining eight articles ranged from 2 to 13 months, with three-quarters of these reporting a delay of between 2–5.3 months. Of the 23 studies defining samples solely by CD, the overall delay ranged from 2 to 26.4 months, with three-quarters of the reported median delays ranging from 2 to 12 months. Finally, from the 16 studies examining UC, median diagnostic delay ranged from 2 to 55.2 months, with three-quarter of studies reporting delay between 2 to 6 months. This overall longer median delay observed in patients with CD compared to UC was also mirrored in the subset of studies where delay had been directly compared between the two conditions within the same populations [5, 7, 13, 21, 23, 34]. Finally, when data for each disease category was arranged by year of publication, the extent of delay remained relatively consistent over time from 2009 to 2021 for IBD and UC. However, for the same time period, CD publications showed greater fluctuations year-on-year in reported extent of delay.

### Factors associated with diagnostic delay

Three studies compared differences in delay related to help-seeking (from symptom onset to primary health care consultation) and that which occurred after the first consultation. Though Vavricka found that delay was significantly greater for CD than UC patients during the help-seeking and first consultation phase, Nguyen only found delay after first consultation to final diagnosis to be significantly greater for CD patients compared to UC patients [6, 14]. Walker et al. [28] reported longer delays across help-seeking, primary care and secondary care in CD over UC (Table 3). Three articles found statistical significance between increasing age and longer diagnostic delay [8, 25, 30]. The study by Foxworthy and Wilson was the only one that reported median values, showing a statistically significant difference in diagnostic delay of CD between patients over 60 (diagnostic delay 16 months) and patients less than 60 years old at diagnosis (diagnostic delay 5 months) [30].

**Table 3:** Extent of delay in inflammatory bowel disease diagnosis at different stages of the patient journey

				Median months of delay (IQR) [range]	
Reason for delay	Reason/point of delay	Author, year	Country	CD	UC
Patient-related delay	Non-help-seeking	Vavricka et al., 2012 [6]	Switzerland	2 (0–6)	1 (0–4)
		Nguyen et al., 2017 [14]	USA	1 (0.2–4.9)	0.7 (0.3–3.0)
		Lee et al., 2017 [12]	South Korea	1.2 (0.5–8.4)	1.9 (0.9–4.9)
		Walker et al., 2020 [28]	UK	3 (0.9–6.7)	2.1 (0.9–3.9)
Healthcare-related delay	Delay following first consultation	Nguyen et al., 2017 [14]	USA	3.5 (1.2–20.5)	1.1 (0.4–5.4)
		Vavricka et al., 2012 [12]	Switzerland	4 (0–18)	1 (0–5)
	Delay from first physician visit to IBD diagnosis	Lee et al., 2017 [12]	South Korea	0.7 (0.1–4.6)	0.2 (0.1–0.6)
	Primary care delay	Walker et al., 2020 [28]	UK	0.3 (0.0–1.2)	0.2 (0.0–0.8)
	Secondary care delay	Walker ﻿et al., 2020 [28]	UK	1.6 (0.6–3.7)	0.9 (0.5–2.0)

A prospective cohort study conducted by Burisch et al. grouped data from 14 Western and 8 Eastern European countries to compare differences in diagnostic delay [9]. For CD, median values of diagnostic delay were 4.6 and 3.4 months, respectively, for Western and Eastern Europe. Diagnostic delay was 2.5 and 2.2 months for UC in Western and Eastern Europe respectively. However, these differences were not statistically different. Finally, Pellino et al. stratified patients with CD by disease behavior and found that only patients receiving a diagnosis of penetrating disease had significantly longer delay compared with other disease behaviors which were non-penetrating (*p* = 0.003) [11] (Table 4).

**Table 4:** Data for additional characteristics of delay

Author, publication year and country	CD/UC	Diagnostic criteria	Comparison	Sample size	% male	Mean age (SEM), range	Diagnostic delay (SD)/range (months)
Foxworthy and Wilson (1985) [30], USA	CD	Chart review	Diagnosis ≥ 60 years old	10	50	68 (1.6), 60–78	Median-16
			Diagnosis < 60 years old	20	35	27 (1.6), 20–45	Median-5
Harper et al. (1986) [8], USA	CD	Clinical/radiologic/endoscopic/operative features	Diagnosis > 64 years old	24	42	57 (2.5)	Mean-76.8 (18)
			Diagnosis < 64 years	24	42	23.3 (2.0)	Mean-28.8 (8.4)
Lin et al.(2016) [25], Taiwan	UC	Not reported	Onset ≥ 60 years old	77	60	68 (6.0), 60–91	Mean-26.4 (54), 0–288
			Onset < 60 years old	459	59	36.8 (11.5), 1–59	Mean-4.8 (9.6), 0–120
Burisch et al. (2014) [9], Europe	CD	Copenhagen diagnostic criteria	Western Europe	430	51	38, 16–89	Median-4.6, 0–372
			Eastern Europe	105	60	32, 15–78	Median-3.4, 0–240
	UC	Copenhagen diagnostic criteria	Western Europe	668	56	39, 15–89	Median-2.5, 0–252
			Eastern Europe	145	57	36, 18–81	Median-2.2, 0–60
Pellino et al. (2015) [15], Italy	CD	Clinical evaluation and a combination of endoscopic, histological, radiological and/or biochemical investigations	Disease behaviour—inflammatory	292	-	-	Median-12, 1–100
			Disease behaviour—stricturing	52	-	-	Median-15, 1–152
			Disease behaviour—penetrating	17	-	-	Median-70, 3–163
			Disease behaviour—global	-	-	-	Median-11, 1–163

*ECCO-ESGAR* European Crohn’s and Colitis Organisation (ECCO) and the European Society of Gastrointestinal and Abdominal Radiology (ESGAR)

## Discussion

This systematic review demonstrates that receiving a prompt diagnosis of IBD remains difficult to achieve, with patients typically experiencing several months of diagnostic delay. In particular, delay is prolonged in patients with CD compared to UC, with the majority of previous studies reporting diagnostic delay less than 12 months for CD, but less than 6 months for UC. Ultimately, these more specific median delay data ranges provide a new benchmark against which interventions to reduce delay in patients with IBD can be compared. However, research examining the specific factors contributing to delay in IBD remains limited and requires further examination to determine any consistent influence.

The skewed nature of diagnostic delay data means that (for the majority) of patients with CD diagnosis is achieved within a year of symptom onset, with three-quarter of patients with UC diagnosed in < 6 months. However, across CD studies in particular, there remains a substantial proportion (one in four studies) where on average, receiving a final diagnosis of CD could take between 12 to 24 months from the initial onset of symptoms. These findings provide a more accurate understanding of the true extent of diagnostic delay and though not intended to minimise the problem of diagnostic delay in UC, the greatest impact in a reduction in delay for patients with IBD may come from focusing on CD.

Though some degree of interval time period between symptom onset and final diagnosis is inevitable and each country must work within its own practical parameters, we believe that delays of less than 6 months or 12 months for all, not just the majority of, UC and CD patients respectively should be strived for. This should certainly be the case for more advanced healthcare systems, such as the UK, where there remains a minority of patients at the extremes who experience excessive delay, as represented by the interquartile ranges (IQR) by Walker et al. (CD 7.6 months median delay [IQR 3.1–15.0]; UC 3.3 months [1.9–7.3]) [28].

Our reported findings are based on the studies included in the sensitivity analysis, rather than all identified studies. By removing the study by Burgmann et al. [5] which reported extremely protracted delays in IBD, CD and UC diagnosis, we have provided a more representative median range for diagnostic delay. Though removal of such outliers is a somewhat arbitrary one, performing this practical sensitivity analysis enabled us to look at the most common length of delay. To support the removal of studies based on their reported, extreme delay values, we examined the details of the study design, but could not identify any clear reason why their data was drastically different to that of the other studies in the systematic review. However, it was of note that this excluded study reported longer diagnostic delay in UC compared to CD, contrary to other included studies.

Possible reasons for UC consistently demonstrating shorter diagnostic delays than CD could be because the location of disease is confined to the large bowel and patients experiencing rectal bleeding may be more likely to present to a doctor [7]. However, CD delays may be longer due to a lack of clinical suspicion and diagnostic testing, associating common CD symptoms like abdominal pain with other conditions such as irritable bowel syndrome and difficulties in identifying disease that is only present in the small bowel [12, 14, 40].

Though the 26 articles which reported overall diagnostic delay were published between 1971 and 2021, the majority was published after 2006 (80%). For the IBD, CD and UC categories, there was no trend of diagnostic delay over this 15-year time period, though the values for IBD and UC were more consistent from 2009 onwards than for CD. However, inherent variations in country demographics, healthcare systems and study designs make such trends difficult to interpret and studies across multiple years in the same countries are needed to examine changes over time in patient delays.

Though overall diagnostic delay in patients with IBD, CD or UC has frequently been reported in the literature, there remains limited data to have examined the role of specific patient or healthcare factors in their impact to the extent of delay experienced. Such sub-analysis has proven useful in other systematic reviews into diagnostic delay, as this provides focus on certain characteristics which could prove to be avenues to reduce delay. For example, a previous systematic review on diagnostic delay in giant cell arteritis found delay was greater in those patients who did not experience cranial symptoms compared to those with cranial symptoms [16]. However, the most frequently examined characteristic we were able to identify was age, and though these studies found delay to be greater in the older of each dichotomized group, there were only three studies, of which two are nearly 40 years old. More studies, of a consistent and repeated design, are needed to make a stronger case for the role of impact of individual characteristics on the extent of diagnostic delay.

Though not reporting the extent of delay associated with a specific characteristic as per our study design, several studies did highlight other characteristics which were considered to have a significant role on delay. Vavricka et al. found that of adults categorized as experiencing ‘longer’ diagnostic delay (CD > 24 months and in UC > 12 months), those aged < 40 years compared to those > 40 years (in contrast to our findings) and who had ileal disease compared colon disease were significantly more likely to experience this prolonged delay [6]. Novacek et al. found ‘greater age’ at diagnosis to be a risk factor for delayed diagnosis in CD as well as in UC. They also found a higher educational level to be a risk factor in patients with CD, but not in UC [7]. Finally, Lee et al. [12] found an association between patients with CD and defined as experiencing ‘long’ diagnostic delay (defined as ≥ 21.4 months and 6.2 months in CD and UC, respectively) and perianal discomfort. The select nature of these samples (e.g. those with ‘long’ delay) make comparison with our own included data difficult.

Diagnostic delay has been explored in many other studies for a variety of conditions, including giant cell arteritis, gynecological cancers and tuberculosis [16, 43, 44]. Exploring the extent of diagnostic delay in medical conditions, where delays are common, is important as it provides a backdrop for future research examining the reasons for prolonged diagnostic delay and potentially inform interventions for reducing delays and improving patient care. As prolonged diagnostic delay of IBD appears to increase the likelihood of complicated disease, reducing delays in IBD diagnosis could improve the clinical outcome of patients with the condition [12, 45]. The specific use of the fecal calprotectin test was not discussed in the studies of this systematic review. It detects levels of calprotectin within stool as a consequence of neutrophil aggregation to the gastrointestinal tract due to active inflammation like that found in IBD [46]. Existing research suggests that this is effective at differentiating between organic and functional origins of bowel disease, thus could reduce delays in IBD diagnosis [47, 48].

The focus on median data within the analysis in this systematic review is a key strength as it reduces problems related to overestimation of averages typically related to use of means from skewed data, providing a more robust estimate of diagnostic delay. Furthermore, as well as examining IBD overall, this systematic review examined the most common disease sub-categories of UC and CD, and, finally, we did not restrict study inclusion based on language, leading to additional studies being included in the review. A limitation of this study is the variation between individual study designs, for instance differences in participant age, method of IBD diagnosis and country. However, despite the introduction of such heterogeneity, this data also provides a fuller picture of the problem of diagnostic delay in this disease group.

In conclusion, this systematic review provides a robust insight into the current extent of diagnostic delay in IBD, indicating that diagnostic delay remains a pertinent issue for patients with IBD, particularly CD, but that the factors which may have a role in delay remain unclear. This systematic review provides a backdrop and benchmark onto which further research can be conducted to reduce the time to IBD diagnosis, particularly exploring knowledge of IBD amongst healthcare professionals and the general population to help reduce overall delay. This future research is particularly important, as reducing diagnostic delay of IBD may improve the clinical course of the disease.

## Supplementary Information

Below is the link to the electronic supplementary material.

Supplementary file1 (PDF 404 KB)

## Declarations

### Disclaimer

The authors are solely responsible for the data and the contents of the paper. In no way, the Honorary Editor-in-Chief, Editorial Board Members, the Indian Journal of Gastroenterology or the printer/publishers are responsible.
