# Supplementary material for: Diagnostic delay in adult inflammatory bowel disease: A systematic review
Source: Indian J Gastroenterol. 2023 Jan 30;42(1):40–52. doi: 10.1007/s12664-022-01303-x (PMC10038954; doi:10.1007/s12664-022-01303-x)
Supplement: Supplementary file 1 — Supplementary file1 (PDF 404 KB) [file 12664_2022_1303_MOESM1_ESM.pdf]

**Supplementary Table 1- Search strategy:**

| Search Strategy | |
|---|---|
| 1 | Exp inflammatory bowel diseases/ |
| 2 | inflammatory bowel disease*.ti,ab,kw. |
| 3 | inflammatory bowel disorder*.ti,ab,kw. |
| 4 | ibd.ti,ab,kw. |
| 5 | crohn*.ti,ab,kw. |
| 6 | ulcerative colitis*.ti,ab,kw. |
| 7 | inflam* colitis*.ti,ab,kw. |
| 8 | colitis*.ti,ab,kw. |
| 9 | colitis/ |
| 10 | 1 OR 2 OR 3 OR 4 OR 5 OR 6 OR 7 OR 8 OR 9 |
| 11 | diagnos* adj3 delay*.ti,ab,kw. |
| 12 | diagnos* adj3 lag*.ti,ab,kw. |
| 13 | diagnos* adj3 interval*.ti,ab,kw. |
| 14 | ((late* or earl*) adj3 diagnos*).ti,ab,kw. |
| 15 | ((late* or earl*) adj3 treat*).ti,ab,kw. |
| 16 | ((late* or earl*) adj3 consult*).ti,ab,kw. |
| 17 | ((late* or earl*) adj3 refer*).ti,ab,kw. |
| 18 | ((late* or earl*) adj3 detect*).ti,ab.kw. |
| 19 | (health* adj3 seek*).ti,ab,kw. |
| 20 | (case* adj3 seek*).ti,ab,kw. |
| 21 | (case* adj3 find*).ti,ab,kw. |
| 22 | (delay* adj3 consult*).ti,ab,kw. |
| 23 | (delay* adj3 detect*).ti,ab,kw. |
| 24 | (delay* adj3 interval*).ti,ab,kw. |
| 25 | (delay* adj3 refer*).ti,ab,kw. |
| 26 | (delay* adj3 treat*).ti,ab,kw. |
| 27 | (delay* adj3 seek*).ti,ab,kw. |
| 28 | Delayed diagnosis/ |
| 29 | Early diagnosis/ |
| 30 | 11 OR 12 OR 13 OR 14 OR 15 OR 16 OR 17 OR 18 OR 19 OR 20 OR 21 OR 22 OR 23 OR 24 OR 25 OR 26 OR 27 OR 28 OR 29 |
| 31 | 10 AND 30 |

**Supplementary Table 2: Quality appraisal of included articles**

| Author and Publication Year | Study design | Selection | | Outcome |
|---|---|---|---|---|
| | | Representativeness of the cohort | Ascertainment of Exposure | Assessment of Outcome |
| Kyle, 1971 | Cross-sectional | Somewhat representative | Secure records | No description |
| Lind, 1985 | Prospective cohort | Somewhat representative | Secure records | Record linkage |
| Foxworthy, 1985 | Prospective cohort | Selected group of users (elderly population) | Secure records | Self-report |
| Harper, 1985 | Cross-sectional | Selected group of users (elderly population) | Secure records | Self-report |
| Langholz, 1991 | Prospective cohort | Somewhat representative | Secure records | Record linkage |
| Munkholm, 1992 | Prospective cohort | Somewhat representative | Secure records | Record linkage |
| Timmer, 1999 | Prospective cohort | Truly representative | Secure records | Record linkage |
| Yang, 2000 | Retrospective & prospective cohort | Truly representative | Secure records | Self-report |
| Burgmann, 2006 | Cross-sectional | Truly representative | Secure records | Self-report |
| Albert, 2008 | Cross-sectional | Somewhat representative | Secure records | No description |
| Romberg-Camps, 2009 | Prospective cohort | Somewhat representative | Secure records | Record linkage |
| Guariso, 2010 | Retrospective & prospective cohort | Somewhat representative | Secure records | Record linkage |
| Vavricka, 2012 | Retrospective cohort | Truly representative | Secure records | Self-report |
| Burisch, 2014 | Prospective cohort | Truly representative | Secure records | Self-report |
| Pellino, 2015 | Cross-sectional | Somewhat representative | Secure records | Record linkage |
| Li, 2015 | Retrospective cohort | Somewhat representative | Secure records | Record linkage |
| Manconi, 2015 | Retrospective cohort | Somewhat representative | Secure records | Self-report |
| Basaranoglu, 2015 | Retrospective cohort | Somewhat representative | Secure records | Record linkage |
| Lin, 2016 | Retrospective cohort | Truly representative | Secure records | Record linkage |
| Lee, 2017 | Retrospective cohort | Somewhat representative | Secure records | Record linkage |
| Cantoro, 2017 | Retrospective cohort | Truly representative | Secure records | Record linkage |
| Nguyen, 2017 | Retrospective cohort | Somewhat representative | Secure records | Record linkage |
| Nahon, 2018 | Retrospective & | Truly representative | Secure records | Record linkage |

| | | | | |
|---|---|---|---|---|
| | prospective cohort | | | |
| Irving, 2018 | Cross-sectional | Truly representative | Secure records | Self-report |
| Szanto, 2018 | Retrospective cohort | Truly representative | Secure records | Record linkage |
| Banerjee, 2018 | Retrospective cohort | Somewhat representative | Secure records | Record linkage |
| Kang, 2019 | Retrospective cohort | Truly representative | Secure records | Record linkage |
| Novacek, 2019 | Cross-sectional | Truly representative | Secure records | Self-report |
| Walker, 2020 | Retrospective cohort | Truly representative | Secure records | Record linkage |
| Gomes, 2021 | Cross-sectional | Truly representative | Secure records | Record linkage |
| Chaparro, 2021 | Prospective cohort | Truly representative | Secure records | Self-report |
